# The 3P‐CP model: Expanding our conceptualization of cancer pain

**DOI:** 10.1002/cncr.70080

**Published:** 2025-09-11

**Authors:** Desiree R. Azizoddin, Jian Zhao, Tamara J. Somers, Sarah E. Taylor, Joseph G. Winger, Susan G. Dorsey, Sarah Orris, Anaanya Nasta, Yael Schenker, Jessica S. Merlin, Heather S. L. Jim, Kristin L. Schreiber, Hailey W. Bulls

**Affiliations:** ^1^ Department of Supportive Oncology Dana Farber Cancer Institute Boston Massachusetts USA; ^2^ Department of Psychiatry Harvard Medical School Boston Massachusetts USA; ^3^ Department of Psychiatry and Behavioral Sciences Duke University School of Medicine Durham North Carolina USA; ^4^ Duke Cancer Institute Duke University Health System Durham North Carolina USA; ^5^ Division of Gynecologic Oncology Department of Obstetrics, Gynecology and Reproductive Sciences University of Pittsburgh Pittsburgh Pennsylvania USA; ^6^ Pain Prevention and Treatment Research Program Department of Psychiatry and Behavioral Sciences Duke University School of Medicine Durham North Carolina USA; ^7^ Department of Pain & Translational Symptom Science University of Maryland School of Nursing University of Maryland Baltimore Maryland USA; ^8^ Challenges in Managing and Preventing Pain (CHAMPP) Clinical Research Center Division of General Internal Medicine Department of Medicine University of Pittsburgh Pittsburgh Pennsylvania USA; ^9^ Section of Palliative Care and Medical Ethics Division of General Internal Medicine Department of Medicine University of Pittsburgh Pittsburgh Pennsylvania USA; ^10^ Palliative Research Center (PaRC) University of Pittsburgh Pittsburgh Pennsylvania USA; ^11^ Department of Health Outcomes and Behavior Moffitt Cancer Center Tampa Florida USA; ^12^ Department of Anesthesiology, Perioperative, and Pain Medicine Brigham and Women’s Hospital Harvard Medical School Boston Massachusetts USA

**Keywords:** cancer pain, conceptual framework, pain management, precision medicine, survivorship

## Abstract

Cancer pain is a complex, multifactorial, and growing public health challenge affecting millions of Americans. Effective pain management is essential for comprehensive cancer care, influencing physical and mental health, quality of life, and functional ability. However, progress in cancer pain management is hindered by the complexity of the issue and a fragmented understanding of the myriad factors shaping the pain experience. Additionally, traditional pain terminology—“acute” (<6 months) and “chronic” pain (≥6 months)—offers limited utility in cancer contexts, highlighting the need for a more nuanced framework. To address this gap, we propose the 3P‐CP model, which conceptualizes cancer pain through three interconnected phases: predisposing factors that increase cancer pain risk, precipitating factors that trigger cancer pain onset, and perpetuating factors that sustain or exacerbate cancer pain over time. This model provides a structured approach to assess the dynamic nature of cancer pain across the entirety of the cancer trajectory. In this paper, key factors associated with each phase of the 3P‐CP model are outlined and their implications for research and clinical care explored. Aligning with the oncology field's shift toward precision medicine, the 3P‐CP model has the potential to guide comprehensive assessment, risk mitigation, prevention, and intervention strategies—supporting efforts to deliver the right targeted and tailored treatments, to the right patients, at the right time.

## Abstract 2

This paper introduces the 3P‐CP model of cancer pain, an innovative framework that highlights the impact of time across the cancer trajectory by identifying predisposing (risk), precipitating (onset), and perpetuating (maintenance) factors. Aligned with the shift toward precision medicine in oncology, the 3P‐CP model offers a valuable approach for developing targeted, personalized pain management plans, guiding implementation at the most effective points in the cancer trajectory.

## INTRODUCTION

A record 2 million Americans are expected to be diagnosed with cancer in 2025, and the majority will report pain during the cancer trajectory (from diagnosis to survivorship, recurrence, and/or end‐of‐life care).^1^, ^2^, ^3^, ^4^ Thanks to improvements in cancer care, most people will live for several years after diagnosis.^5^ However, the long‐term management of common cancer sequelae, including pain, present a new series of challenges for survivors and clinicians alike. The consequences associated with unmanaged cancer pain are clear: increased psychological distress, impaired physical functioning, reduced quality of life, and higher health care utilization and costs, among others.^6^, ^7^, ^8^, ^9^ Thus, treating cancer pain is an important component in cancer care.

A key challenge in cancer pain management is accurately capturing the complex relationship between acute and chronic pain over time. In nonmalignant settings, pain onset is frequently associated with a specific precipitating event (e.g., injury).^1^, ^10^ Though symptoms may wax and wane, pain reported within 3 to 6 months is typically considered to be “acute pain” related to tissue injury, whereas pain lasting 6+ months is “chronic pain” and is attributed to centralized pain mechanisms.^11^ This terminology is of limited utility in cancer, which is characterized by unpredictable and complex disease progressions that complicate the delineation of “acute” and “chronic” periods. Adding to the challenge, cancer disease pathophysiology changes in response to progression or treatment response, while cancer treatments themselves are a common contributor to significant pain—especially when repeated over weeks, months, or years.^12^ Practically, this causes a cascade of several “acute” pain episodes concurrently, likely underscored by increasing centralized “chronic” mechanisms over time.

Significant efforts have been made to develop systematic, evidence‐based conceptualizations of pain.^13^ Perhaps the most defining conceptual framework is Engel’s 1977 biopsychosocial model, which asserted that psychological and social factors contribute to the experience of pain alongside biomedical factors.^14^, ^15^ “Total pain,” first described by Dame Cicely Saunders, is another concept frequently used in palliative care and hospice settings that integrates spiritual distress alongside biological, psychological, and social pain influences.^16^ In more recent years, the ACTTION‐American Pain Society Pain Taxonomy created a multidimensional framework for “chronic” pain classification that emphasizes five key dimensions: (1) diagnostic criteria; (2) features; (3) medical comorbidities, (4) neurobiological, psychosocial, and functional consequences; and (5) mechanisms, risk factors, and protective factors.^17^ Subsequent work produced a similar framework focused on “acute” pain, including for postsurgical breast cancer pain.^11^, ^18^ However, adoption and application of these existing taxonomies to cancer pain in a way that can guide more nuanced clinical pain management has been limited, and the complexity of cancer pain remains difficult to fully conceptualize.

Spielman’s 3P model (1987) may help characterize distinct phases of pain onset and maintenance and allow time‐varying influences to occur in the cancer trajectory.^19^ Predisposing factors are characteristics that place patients at higher risk for developing cancer pain, whereas precipitating factors are clinical factors that initiate the onset of cancer pain. Perpetuating factors are characteristics and behaviors that maintain cancer pain, leading to persistent symptoms. The 3P model was first proposed to describe chronic insomnia; it has since been applied to various diseases and symptoms, including cancer‐related fatigue, chronic noncancer pain, and chemotherapy‐induced peripheral neuropathy.^20^, ^21^, ^22^ Proponents of the 3P model highlight the potential to build on broad, foundational biopsychosocial concepts with increased specificity; the ability to place factors in time, illuminating assessment and intervention points; and the relative ease of operationalizing this model to inform targeted, tailored interventions. Importantly, a 3P model of cancer pain can support a move toward precision medicine, facilitating the delivery of the right interventions to the right people at the right time.

Here, we propose the application of Spielman’s 3P framework to cancer pain (3P‐CP). The 3P‐CP model is intended to be hypothesis‐generating, providing an example of the ways this model can be used to conceptualize the development and maintenance of cancer pain. We define key components of the 3P model, apply relevant cancer pain concepts in each phase, and comment on implications for future research and clinical efforts.

### The 3P‐CP: Application of the 3P model to cancer pain

Common approaches to framework development include adapting existing frameworks, evaluating existing literature conceptually related to the central concept, and integrating transdisciplinary expert opinions from consensus statements, systematic reviews, and peer‐reviewed commentaries.^23^, ^24^, ^25^ Thus, we began with a broad review of frameworks and diagnostic criteria commonly used to conceptualize cancer symptomatology. From this literature, we selected Spielman’s 3P model as a guide.

The next step in the process was to evaluate key basic science, translational, and clinical contributors to cancer pain. The literature base is extensive, and we considered conducting a formal systematic review. However, we deemed this approach inappropriate because we did not intend to quantify evidence on a specific research question. Rather, our goal was to summarize key concepts as exemplars in the 3P‐CP model, more consistent with a narrative review. Narrative reviews are rigorous yet flexible, making them ideally suited for broad and complex topics.^26^ Although any publication detailing contributors to and consequences of cancer pain was eligible for inclusion in the manuscript, we emphasized review of clinical guidelines, topical and systematic reviews, and recent publications describing innovations in cancer pain management.

In the final step, we proposed key factors related to the development and maintenance of cancer pain and adapted the guiding framework to reflect these factors, resulting in the 3P‐CP. We gathered a transdisciplinary group of coauthors with clinical and research expertise in cancer pain and presented the 3P‐CP to them for feedback regarding proposed concepts and additional factors for integration. The 3P‐CP model was revised and refined according to coauthor feedback. We organized our findings by describing proposed contributors to each component, then potential implications and interventions at each phase.

**FIGURE 1:**
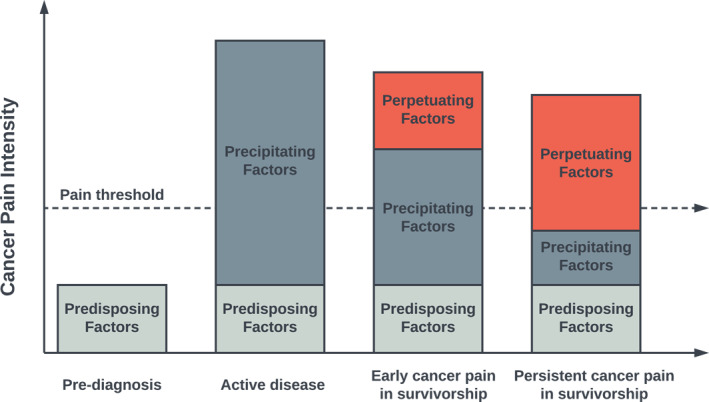
3P‐CP model, illustrating one cycle of cancer pain development and maintenance. 3P‐CP indicates the 3P framework to cancer pain.

**FIGURE 2:**
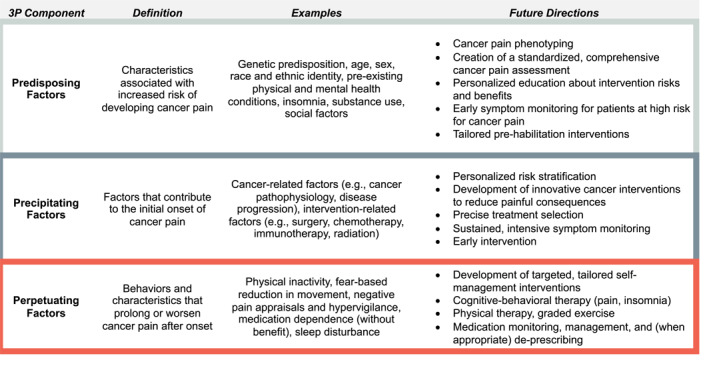
3P definitions, examples, and future directions.

**FIGURE 3:**
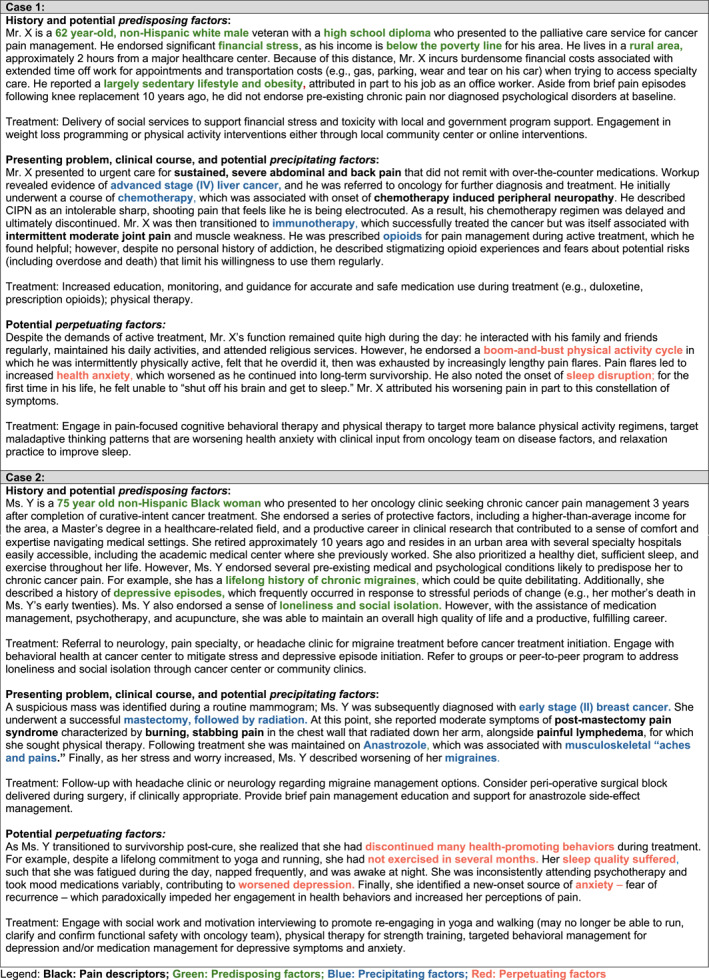
Example case conceptualizations.

A visual depiction of the 3P‐CP model is presented in Figure 1 and is summarized in Figure 2. Example case conceptualizations are shown in Figure 3.

### Predisposing factors

Predisposing factors are baseline characteristics associated with increased risk of cancer pain. Assessment of predisposing factors may be appropriate at diagnosis and/or before cancer‐directed treatments (e.g., surgery, chemotherapy, radiation). There is a large scientific literature on predisposing risk factors for cancer pain.^27^, ^28^, ^29^, ^30^ Briefly, previously identified biological factors may include genetic predispositions, age, race and ethnic identity, sex, preexisting chronic pain conditions unrelated to cancer (e.g., sciatic pain, fibromyalgia, osteoarthritis), and other clinical comorbidities (e.g., obesity, rheumatological, immune conditions),^31^, ^32^, ^33^, ^34^ whereas psychological contributors include preexisting trauma, anxiety, depression, pain‐related cognitions (e.g., catastrophizing), insomnia, and substance use behavior (e.g., smoking, alcohol use).^6^, ^35^, ^36^, ^37^ Relevant predisposing social factors may include lack of access to medical care, experiences with stigma and discrimination, rurality, lower socioeconomic status, and lack of caregiving support. Existing evidence typically focuses on individual factors or clusters, and relatively little is known about how predispositions may interact.^6^ Further investigation of predisposing factors in a comprehensive model can help to identify the most impactful combinations of risk factors, illuminate important underlying mechanisms, and build risk assessment tools.

Relatively fixed characteristics like age, racial and ethnic identity, and biological sex will not be direct therapeutic targets. Rather, these factors may inform the development of risk profiles, offering avenues to understand why these patients are particularly vulnerable. For example, a stable predisposing factor—biological sex—may influence pain via estrogen and progesterone fluctuations commonly higher in females, which sensitizes pain pathways and increases pain sensitivity.^32^, ^38^ This information can be used to develop preventative treatments that target underlying mechanisms (e.g., hormonal fluctuations, menopause), recruit participants who meet relevant risk criteria (e.g., women), and deliver interventions to participants who are most likely to be at risk and thus benefit. Clinically, a better understanding of stable predisposing factors can provide personalized education about treatment risks and benefits while justifying earlier, more intensive symptom monitoring and management for patients predisposed to severe and/or persistent pain.

Other predisposing factors lend themselves more readily to direct intervention. Proactive management of modifiable predisposing factors (e.g., comorbid conditions) can decrease the likelihood of developed shared underlying pathways with cancer pain and creating exponentially worse symptoms. For example, interventions to improve obesity—commonly associated with increased pain sensitivity—can reduce inflammation and mechanical strain while improving depression and sleep quality, potentially mitigating the impact of cancer pain before it even starts.^39^, ^40^ Similarly, preexisting experiences with pain are known to influence pain severity reports in both laboratory settings^41^ and clinical populations (e.g., postoperative pain).^42^, ^43^, ^44^ This relationship is thought to be facilitated by sensitized central mechanisms, exacerbating the intensity of each subsequent exposure. Treating preexisting sources of pain may slow this process, reducing the severity and longevity of cancer pain episodes.

From a psychological perspective, it is well established that pain and mood bidirectionally influence one another, with evidence to suggest that baseline anxiety and depression have leading influences on subsequent pain experiences.^15^, ^45^, ^46^, ^47^ Thus, promoting improved psychological health early on during cancer treatment onset such as disrupting negative thoughts that perpetuate low mood and reinforce sedentary behaviors may mitigate the wide‐ranging impacts of cancer pain preventing worsened pain outcomes over time. In addition to general depression and anxiety treatments (e.g., antidepressants),^48^, ^49^ potential interventions including detailed, brief psychoeducation^50^, ^51^; behavioral self‐management and coping skills^52^; and proactive connection to important support resources (e.g., behavioral health care, support groups) could be targeted specifically for those with greater relative risk earlier in treatment, limiting the impact of depressive/anxiety symptoms on cancer pain incidence or severity early on.

Finally, predisposing social factors provide opportunities for risk mitigation. Clinicians and social workers can partner to educate patients about health‐promoting resources, facilitate supportive care screening, identify resources to offset financial toxicity, and advocate for increased access in underserved areas.^53^, ^54^ Additionally, social support—particularly within close relationships—can influence pain outcomes. Partner or parental responses to pain (e.g., validation, distress) can shape patients’ pain trajectories and coping strategies through meaningful caregiver interventions.^55^, ^56^ Taken together, addressing predisposing factors early in the cancer trajectory can help patients start from the best possible place, mitigating or preventing the wide‐ranging impacts of cancer pain.

### Precipitating factors

Precipitating factors contribute to the initial onset of cancer pain, including disease progression and tumor invasion, surgical intervention, chemotherapy, or radiation treatment. Precipitating factors exert their influence through multiple overlapping, pathophysiological processes rather than a single source, reflecting complex interactions between cancer pathophysiology, pain mechanisms, and treatment consequences.^57^ Oxidative stress,^58^ neuronal sensory damage,^59^ abnormal activation of pain fibers,^60^ increased proinflammatory cytokines,^61^, ^62^ peripheral and spinal epigenetic changes,^63^ and central sensitization^61^ are all believed to contribute to the onset and early maintenance of cancer pain. The precipitating phase of the 3P‐CP is most analogous to “acute” pain episodes in other pain etiologies. Unfortunately, patients frequently undergo varied and repeated precipitating factors throughout cancer treatment, essentially restarting the timeline and reinforcing underlying pain mechanisms each time.

Severe cancer pain can disrupt treatment and compromise key clinical outcomes (e.g., treatment response, survival). Thus, effectively identifying and intervening on cancer‐related precipitating factors is essential to maximizing treatment success.^1^ An example of a particularly challenging condition is the painful subtype of chemotherapy‐induced peripheral neuropathy (CIPN), a dose‐limiting side effect of chemotherapy: burning and tingling sensations and at times, pain.^17^, ^64^, ^65^, ^66^, ^67^ Here, chemotherapy is the precipitating factor, influenced by the chemotherapeutic agent used, dose per cycle, cumulative dose, among other characteristics. There are no evidence‐based strategies for CIPN prevention and only one Food and Drug Administration–approved medication exists (duloxetine).^65^, ^68^, ^69^ Thus, the primary management strategy for CIPN is to delay chemotherapy, change dose regimens, or discontinue chemotherapy entirely. These necessary but unwanted deviations threaten the ultimate goal of curative‐intent treatment: survival.

Identification of key precipitating factors can influence cancer care in two important ways. First, understanding which components of cancer‐directed treatments result in the worst symptoms (e.g., types of chemotherapy associated with severe, painful CIPN, surgical interventions with highest pain risk) can offer clear priorities for improvement. The oncology field has demonstrated remarkable capacity for innovative interventions that directly improve clinical outcomes. It is imperative to continue innovating toward treatments that are less painful while retaining efficacy. Second, understanding how predisposing and precipitating factors interact will facilitate the discovery of targeted, tailored treatment plans early in the cancer trajectory. In some settings (e.g., breast cancer), recognition of interactive predisposing and precipitating factors has already proven impactful. In perioperative settings, evidence suggests that implementation of enhanced recovery after surgery protocols that emphasize transdisciplinary care before, during, and after surgery can result in improved postsurgical pain.^70^, ^71^, ^72^ However, many patients still go on to develop chronic pain after mastectomy.^28^ Interestingly, moderation analysis revealed differential efficacy of preventive analgesic strategies, suggesting that patients with relatively higher risk may derive more benefit from analgesic strategies than others.^70^ Further exploration of key predisposing factors can help to match characteristics and update preventive analgesic protocols to maximize benefit.

In the near term, research to develop personalized risk assessment tools that integrate patient‐, cancer‐, and treatment‐level data can help clinicians anticipate outcomes over the course of treatment (e.g., who is most likely to develop pain) and intervene accordingly (e.g., early pain screening and initiating preventive strategies such as selecting lower neurotoxic chemotherapies for high‐risk patients, delivering intraoperative blocks/medications,^73^ presurgical education,^52^ increased postsurgical monitoring^71^, ^72^). In cases where there is more than one intervention available, this information may also help clinicians select the best treatment recommendation for the patient’s unique risk profile. This approach is likely to have economic benefits, minimizing unplanned and emergent health care utilization for patients with unmanaged pain.^74^ Outcomes should be studied to determine the risks and benefits of personalized oncology tools that integrate symptom data, like pain, in their predictions. In the long term, leveraging predisposing and precipitating factors early in the cancer trajectory can be a powerful tool in proactively avoiding the transition to persistent, intractable cancer pain.

### Perpetuating factors

Perpetuating factors are characteristics and behaviors that prolong or worsen cancer pain after onset. Patients may self‐manage cancer pain in ways that help initially, including resting more, moving less, vigilance to symptom intensity, and/or using prescription opioids. As the pain continues, patient concerns about the meaning of their pain may shift to fear of recurrence, for example, or worsening disability, which further amplifies autonomic reactivity and discourages functional movement. Over time, short‐term management strategies can become entrenched patterns that compromise physical and mental health, decrease strength, reduce function, and ultimately reinforce cancer pain.^75^, ^76^, ^77^

Fortunately, perpetuating factors are frequently modifiable. An apt example is physical inactivity, in which patients may reduce their activity levels in an attempt to reduce pain earlier in the pain experience. As time progresses, patients may find themselves with increasingly negative pain appraisals and recurrent fears of movement, leading to pain hypervigilance and avoidance of activity, which in turn results in physical decompensation and disability, making subsequent activity more painful than ever (the “fear‐avoidance cycle”).^15^, ^78^ In this case, there are existing evidence‐based interventions that can be deployed to improve physical activity and pain, including physical therapy, graded exercise, and/or cognitive‐behavioral therapy with input from their oncology team.^76^, ^79^, ^80^, ^81^

Sustained opioid use over time may present as a unique perpetuating factor for cancer pain. Disentangling the relationship between pain and prescription opioids is challenging in this population because opioids are guideline‐concordant and frequently used during active disease. However, sustained prescription opioid use may paradoxically increase pain sensitivity through opioid‐induced hyperalgesia.^82^, ^83^, ^84^, ^85^ Though mechanisms are poorly understood, opioid‐induced hyperalgesia is attributed to changes in underlying neural, molecular, and chemical changes that increase nociception. As patients engage in recommendations believed to be helpful, such as taking prescription opioids, this may inadvertently increase their risk for persistent, unremitting pain and reduced quality of life into survivorship.^86^, ^87^, ^88^ This may be especially true for patients receiving unimodal opioid treatment without concurrent transdisciplinary pain care.^1^, ^89^ Additionally, long‐term cancer pain treatment is complicated by the lack of a clear “home” for opioid management in survivorship, relative dearth of evidenced‐based clinical guidelines, and stigma surrounding prescription opioids, which hinders critical prescribing discussions between clinicians and with patients directly.^86^, ^87^, ^90^, ^91^, ^92^ Research is critically needed to characterize the potential risks of sustained opioids across the entire cancer trajectory.

### Characterizing dynamic factors across the cancer trajectory

The 3P‐CP attempts to place key factors at the point in time where they may be the most important to inform further scientific evaluation and/or intervention efficacy, based on what is known about these factors. However, 3P‐CP factors may have distinct features at different points of the cancer trajectory with unique implications for intervention. For example, consider the relationship between sleep and pain.^93^ It is widely appreciated that poor sleep quality at baseline has a unidirectional, worsening influence on subsequent pain in rodent and animal models. Persistent sleep disturbance can predispose the nervous system to heightened pain sensitivity and lower pain tolerance through a variety mechanisms, including impaired central nervous system recovery, increased pro‐inflammatory cytokine activity, and dysregulated hypothalamic‐pituitary‐adrenal axis function.^15^, ^94^, ^95^ These biological changes can amplify nociceptive signaling and central sensitization, making the incidence of pain following surgery or treatment more likely and worsening pain severity over time.^96^ Thus, if a patient presents with preexisting sleep disturbance, it would be considered a predisposing factor for the development of future cancer pain. Consequently, offer sleep interventions at the very beginning of treatment to reduce the risk of cancer pain onset would be especially important. However, cancer treatment is burdensome, and even patients with excellent baseline sleep may experience disruptions throughout treatment. The reinforcement of persistent central nervous system reactivity, combined with behavioral responses to sleep loss—such as reduced physical activity, reliance on maladaptive coping strategies, worsened mood, and increased stress—may further exacerbate this vicious sleep–pain cycle. In this scenario, sleep would be labeled as a perpetuating factor for cancer pain, requiring repeated assessment and intervention later in the cancer trajectory. Similarly, previous depressive episodes (predisposing factors) before cancer treatment may increase one’s risk for developing pain after treatment because of underlying neuro‐inflammatory and behavioral processes. In contrast, initial depressive episodes may occur after the onset of cancer pain, especially in the setting of physical inactivity, poor coping skills, and isolation (perpetuating factors).

### Implications

The 3P‐CP builds on foundational work in the fields of pain, oncology, and behavioral medicine, offering an innovative conceptualization of cancer pain that recognizes the influence of time across the cancer trajectory. Pain associated with cancer and its treatment is a significant, growing public health problem, with an estimated 18 million cancer survivors in the United States.^5^, ^97^ Although advances in cancer treatment have led to longer survival—a major success in the science and clinical practice of oncology—this translates to even more patients living with pain during and long after treatment.^98^ There have been substantial efforts to identify contributors to and consequences of cancer pain, providing a strong evidence base with which to devise the 3P‐CP model. Integrating this foundational research into a comprehensive, interdisciplinary approach that encompasses cancer pain’s multifaceted complexities can help facilitate personalized and effective cancer pain care. Adoption of the 3P‐CP model could be a key component in addressing this pressing clinical need by creation of a comprehensive pain assessment, by facilitating prevention efforts via phenotyping, by advancing the development and delivery of targeted, tailored interventions, and by helping clinicians integrate cutting‐edge research into their clinical practice

First, the 3P‐CP model attempts to organize complexity across the cancer trajectory, deepening our understanding of what factors matter and when, to develop meaningful symptom assessments across the cancer trajectory. Future efforts should create a comprehensive assessment of predisposing factors at baseline, document clinical and treatment‐related precipitating factors, and track perpetuating factors in real time to understand exactly when these behaviors and characteristics come online. Integration of validated monitoring tools, such as ecological momentary assessments, patient‐reported outcome tools, and actigraphy data, will provide critical real‐world data reflecting patient behavior outside of the clinic.^99^, ^100^ Key findings can then be used to iteratively refine the 3P‐CP model and update standard assessment strategies.

A second use of the 3P‐CP is to support cancer pain prevention efforts, which has been a topic of considerable interest with limited progress because of the vast complexity of time‐varying influences on cancer pain. Cutting‐edge, deep phenotyping methods now offer a sophisticated strategy to analyze a high volume of biopsychosocial information, clinical and sociodemographic characteristics, biomarker profiles, neuroimaging, ecological momentary assessments, and wearable sensor data.^101^, ^102^ Importantly, deep phenotyping reaches beyond simple individual associations to provide insight into how biopsychosocial factors relate to each other, providing a powerful tool to evaluate key variables in disease progression and treatment response. This approach is increasingly used in models of health and disease, including pain of other etiologies.^103^, ^104^, ^105^, ^106^, ^107^ With the 3P‐CP model as a guide, phenotyping efforts may begin to disentangle the complex threads of cancer pain development and maintenance. Such findings can be used to provide personalized risk education and treatment recommendations, alongside efforts to develop targeted interventions at important points along the cancer trajectory.

A final use of the 3P‐CP model is to guide intervention research efforts, especially when conducted longitudinally. Current cancer pain treatments have a high level of response variability, suggesting that additional information is needed to inform intervention development and treatment selection. Successful matching of patient phenotypes and interventions can maximize benefit and reduce harms, representing a significant improvement in cancer pain management. The 3P‐CP can also encourage researchers developing new interventions to think longitudinally about how and when interventions work, optimizing the delivery and implementation of these efforts. For example, predisposing factors can identify patients at high risk for cancer pain early in the cancer trajectory, when effective interventions may prevent the onset of cancer pain entirely. Clinical and treatment‐related precipitating factors present opportunities for improving oncology care, including selection of treatments with maximum benefit and minimum risk; tailored perioperative strategies for enhanced recovery; optimized pharmacologic and interventional pain treatments; and paired “prehabilitation” programs to reduce pain severity and longevity during treatment. Perpetuating factors offer the opportunity to deliver self‐management and cognitive‐behavioral therapies, maximize pro‐health behaviors, and reduce the burden of persistent cancer pain throughout the cancer trajectory.

### Advancing the 3P‐CP: Opportunities for refinement

The 3P‐CP is intended to provide a novel organizing structure for conceptualizing cancer pain. It is intended to be flexible and responsive to new research findings. For example, literature examining specific contributors to and consequences of cancer pain is extensive, yet frequently focused on single clinical presentations (e.g., primary cancer site) and/or setting. The proposed 3P‐CP attempts to identify common factors broadly associated with cancer pain and situate them in time, pulling from a variety of cancer types; yet, this list of potential factors is not exhaustive, and they may differ across cancer disease, treatment type, and cancer pain syndromes. Additionally, factors described here likely interact with disease pathology, underlying central nervous system reactivity, and psychophysiological processes in ways that are not yet fully understood. There are important ongoing efforts to understand pathways that link these processes, and future discoveries will be key for refining iterations of the 3P‐CP model. This information will inform clinical applications of the 3P‐CP, helping to develop targeted, tailored cancer pain interventions.

## CONCLUSION

It is broadly recognized that cancer pain is a multifactorial, complex, and growing public health challenge. The 3P‐CP offers an innovative way to conceptualize cancer pain for both patients and providers in a more comprehensive manner by moving past “acute” and “chronic” terminology to a model that integrates complex, time‐varying pain contributors across the cancer trajectory including predisposing, precipitating, and perpetuating factors. Application of the 3P‐CP model can support the development of comprehensive pain assessments across key points along the cancer trajectory, identify cancer pain phenotypes that confer critical information about pain risk and likely outcomes, and guide longitudinal intervention research to deliver the right interventions to the right person, at the right time. Importantly, the 3P‐CP model can be used to define patients’ complex cancer pain experiences and inform the use of overlapping pain treatments to provide clarity for both patients and their clinicians on how to improve the complex, highly burdensome symptom of cancer pain. Consistent with the oncology field’s movement toward precision medicine, we expect that the 3P‐CP could be a key component in the creation of targeted, tailored pain management plans for patients with cancer, informing implementation at the most beneficial points in the cancer trajectory.

## AUTHOR CONTRIBUTIONS

**Desiree R. Azizoddin:** Conceptualization; investigation; methodology; data curation and analysis; writing—original draft; and writing—review and editing. **Jian Zhao:** Conceptualization; investigation; methodology; data curation and analysis; writing—original draft; and writing—review and editing. **Tamara J. Somers:** Conceptualization and writing—review and editing. **Sarah E. Taylor:** Conceptualization and writing—review and editing. **Joseph G. Winger:** Conceptualization and writing—review and editing. **Susan G. Dorsey:** Conceptualization and writing—review and editing. **Sarah Orris:** Conceptualization and writing—review and editing. **Anaanya Nasta:** Conceptualization and writing—review and editing. **Yael Schenker:** Conceptualization and writing—review and editing. **Jessica S. Merlin:** Conceptualization and writing—review and editing. **Heather S. L. Jim:** Conceptualization and writing—review and editing. **Kristin L. Schreiber:** Conceptualization; investigation; methodology; data curation and analysis; writing—original draft; and writing—review and editing. **Hailey W. Bulls:** Conceptualization; investigation; methodology; data curation and analysis; supervision; writing—original draft; and writing—review and editing.

## CONFLICT OF INTEREST STATEMENT

The authors declare no conflicts of interest.

## Data Availability

Data sharing not applicable to this article as no datasets were generated or analyzed during the current study

## References

[1] Kwon JH . Overcoming barriers in cancer pain management. J Clin Oncol. 2014;32(16):1727‐1733. doi:10.1200/jco.2013.52.4827 24799490

[2] Jones KF , Magee LW , Fu MR , et al. The contribution of cancer‐specific psychosocial factors to the pain experience in cancer survivors. J Hospice Palliat Nurs. 2023;25(5):E85‐E93. doi:10.1097/njh.0000000000000965 PMC10524730 37402212

[3] American Cancer Society . Cancer Facts & Figures 2025. Accessed March 7, 2025. https://www.cancer.org/cancer‐facts‐and‐statistics

[4] Paice JA , Portenoy R , Lacchetti C , et al. Management of chronic pain in survivors of adult cancers: American Society of Clinical Oncology Clinical Practice Guideline. J Clin Oncol. 2016;34(27):3325‐3345. doi:10.1200/jco.2016.68.5206 27458286

[5] Tonorezos E , Devasia T , Mariotto AB , et al. Prevalence of cancer survivors in the United States. JNCI. 2024;116(11):1784‐1790. doi:10.1093/jnci/djae135 39002121 PMC11542986

[6] Zaza C , Baine N . Cancer pain and psychosocial factors: a critical review of the literature. J Pain Symptom Manag. 2002;24(5):526‐542. doi:10.1016/s0885-3924(02)00497-9 12547052

[7] O'Mahony S , Goulet J , Kornblith A , et al. Desire for hastened death, cancer pain and depression: report of a longitudinal observational study. J Pain Symptom Manag. 2005;29(5):446‐457. doi:10.1016/j.jpainsymman.2004.08.010 15904747

[8] Caterino JM , Adler D , Durham DD , et al. Analysis of diagnoses, symptoms, medications, and admissions among patients with cancer presenting to emergency departments. JAMA Netw Open. 2019;2(3):e190979. doi:10.1001/jamanetworkopen.2019.0979 30901049 PMC6583275

[9] Azizoddin DR , Beck M , Flowers KM , et al. Psychological evaluation of patients with cancer presenting to the emergency department with pain: independent predictors of worse pain severity, interference, and higher hourly opioid administration. JCO Oncology Practice. 2022;18(10):e1648‐e1660. doi:10.1200/op.22.00142 35994699 PMC9810145

[10] Grichnik KP , Ferrante FM . The difference between acute and chronic pain. Mt Sinai J Med. 1991;58(3):217‐220. 1875958

[11] Kent ML , Tighe PJ , Belfer I , et al. The ACTTION–APS–AAPM Pain Taxonomy (AAAPT) multidimensional approach to classifying acute pain conditions. Pain Med. 2017;18(5):947‐958. doi:10.1016/j.jpain.2017.02.421 28482098 PMC5431381

[12] Haroun R , Wood JN , Sikandar S . Mechanisms of cancer pain. Frontiers Pain Res. 2023;3:1030899. doi:10.3389/fpain.2022.1030899 PMC9845956 36688083

[13] Caraceni A , Shkodra M . Cancer pain assessment and classification. Cancers. 2019;11(4):510. doi:10.3390/cancers11040510 30974857 PMC6521068

[14] Engel GL . The need for a new medical model: a challenge for biomedicine. Science. 1977;196(4286):129‐136. doi:10.1126/science.847460 847460

[15] Gatchel RJ , Peng YB , Peters ML , Fuchs PN , Turk DC . The biopsychosocial approach to chronic pain: scientific advances and future directions. Psychol Bull. 2007;133(4):581‐624. doi:10.1037/0033-2909.133.4.581 17592957

[16] Richmond C . Dame Cicely Saunders. Biography. BMJ. 2005;331(7510):238.

[17] Fillingim RB , Bruehl S , Dworkin RH , et al. The ACTTION‐American Pain Society Pain Taxonomy (AAPT): an evidence‐based and multidimensional approach to classifying chronic pain conditions. J Pain. 2014;15(3):241‐249. doi:10.1016/j.jpain.2014.01.004 24581634 PMC4454364

[18] Schreiber KL , Belfer I , Miaskowski C , Schumacher M , Stacey BR , Van De Ven T . AAAPT diagnostic criteria for acute pain following breast surgery. J Pain. 2020;21(3‐4):294‐305. doi:10.1016/j.jpain.2019.08.008 31493489 PMC8290886

[19] Spielman AJ , Caruso LS , Glovinsky PB . A behavioral perspective on insomnia treatment. Psychiatr Clin North Am. 1987;10(4):541‐553. doi:10.1016/s0193-953x(18)30532-x 3332317

[20] Sleight AG , Crowder SL , Skarbinski J , et al. A new approach to understanding cancer‐related fatigue: leveraging the 3P model to facilitate risk prediction and clinical care. Cancers (Basel). 2022;14(8):1982. doi:10.3390/cancers14081982 35454890 PMC9027717

[21] Lee KT , Bulls HW , Hoogland AI , James BW , Colon‐Echevarria CB , Jim HSL . Chemotherapy‐induced peripheral neuropathy (CIPN): a narrative review and proposed theoretical model. Cancers (Basel). 2024;16(14):2571. doi:10.3390/cancers16142571 39061210 PMC11274737

[22] Wright CD , Tiani AG , Billingsley AL , Steinman SA , Larkin KT , McNeil DW . A framework for understanding the role of psychological processes in disease development, maintenance, and treatment: the 3P‐disease nodel. Hypothesis and theory. Front Psychol. 2019:10doi. doi:10.3389/fpsyg.2019.02498 31824367 PMC6879427

[23] Bulls HW , Chu E , Goodin BR , et al. Framework for opioid stigma in cancer pain. Pain. 2022;163(2):e182‐e189. doi:10.1097/j.pain.0000000000002343 34010940 PMC8589872

[24] Hooten WM , Brummett CM , Sullivan MD , et al. A conceptual framework for understanding unintended prolonged opioid use. Mayo Clin Proc. 2017;92(12):1822‐1830. doi:10.1016/j.mayocp.2017.10.010 29108841

[25] Merlin JS , Zinski A , Norton WE , et al. A conceptual framework for understanding chronic pain in patients with HIV. Pain Pract. Mar 2014;14(3):207‐216. doi:10.1111/papr.12052 23551857

[26] Sukhera J . Narrative reviews: flexible, figorous, and practical. J Grad Med Educ. 2022;14(4):414‐417. doi:10.4300/jgme-d-22-00480.1 35991099 PMC9380636

[27] Schreiber KL , Zinboonyahgoon N , Xu X , et al. Preoperative psychosocial and psychophysical phenotypes as predictors of acute pain outcomes after breast surgery. J Pain. 2019;20(5):540‐556. doi:10.1016/j.jpain.2018.11.004 30476655 PMC6511455

[28] Schreiber KL , Zinboonyahgoon N , Flowers KM , et al. Prediction of persistent pain severity and impact 12 months after breast surgery using comprehensive preoperative assessment of biopsychosocial pain modulators. Ann Surg Oncol. 2021;28(9):1‐24. doi:10.1245/s10434-020-09479-2 PMC8280248 33452600

[29] Dalton JA , Higgins MK , Miller AH , Keefe FJ , Khuri FR . Pain intensity and pain interference in patients with lung cancer: a pilot study of biopsychosocial predictors. Am J Clin Oncol. 2015;38(5):457‐464. doi:10.1097/coc.0b013e3182a79009 24064756 PMC3962526

[30] Leysen L , Beckwée D , Nijs J , et al. Risk factors of pain in breast cancer survivors: a systematic review and meta‐analysis. Support Care Cancer. 2017;25(12):3607‐3643. doi:10.1007/s00520-017-3824-3 28799015

[31] Yang GS , Barnes NM , Lyon DE , Dorsey SG . Genetic variants associated with cancer pain and response to opioid analgesics: implications for precision pain management. Semin Oncol Nurs. 2019;35(3):291‐299. doi:10.1016/j.soncn.2019.04.011 31085105 PMC6688486

[32] Mogil JS , Parisien M , Esfahani SJ , Diatchenko L . Sex differences in mechanisms of pain hypersensitivity. Neurosci Biobehav Rev. 2024;163:105749. doi:10.1016/j.neubiorev.2024.105749 38838876

[33] Yoon SL , Scarton L , Duckworth L , et al. Pain, symptom distress, and pain barriers by age among patients with cancer receiving hospice care: comparison of baseline data. J Geriatr Oncol. 2021;12(7):1068‐1075. doi:10.1016/j.jgo.2021.04.008 33967022 PMC8429256

[34] Beck M , Schreiber KL , Wilson JM , et al. A secondary analysis: the impact of pre‐existing chronic pain among patients with cancer presenting to the emergency department with acute pain. Support Care Cancer. 2024;32(2):129. doi:10.1007/s00520-024-08314-8 38270721 PMC11069411

[35] McCowat M , Fleming L , Vibholm J , Dixon D . The psychological predictors of acute and chronic pain in women following breast cancer surgery: a systematic review. Clin J Pain. 2019;35(3):261‐271. doi:10.1097/ajp.0000000000000672 30531400

[36] Giusti EM , Lacerenza M , Manzoni GM , Castelnuovo G . Psychological and psychosocial predictors of chronic postsurgical pain: a systematic review and meta‐analysis. Pain. 2021;162(1):10‐30. doi:10.1097/j.pain.0000000000001999 32694386

[37] Unseld M , Zeilinger EL , Fellinger M , et al. Prevalence of pain and its association with symptoms of post‐traumatic stress disorder, depression, anxiety and distress in 846 cancer patients: a cross sectional study. Psychooncology. 2021;30(4):504‐510. doi:10.1002/pon.5595 33210393 PMC8049050

[38] Fillingim RB , King CD , Ribeiro‐Dasilva MC , Rahim‐Williams B , Riley JL, III . Sex, gender, and pain: a review of recent clinical and experimental findings. J Pain. 2009;10(5):447‐485. doi:10.1016/j.jpain.2008.12.001 19411059 PMC2677686

[39] McVinnie DS . Obesity and pain. Br J Pain. 2013;7(4):163‐170. doi:10.1177/2049463713484296 26516520 PMC4590160

[40] Okifuji A , Hare BD . The association between chronic pain and obesity. J Pain Res. 2015;8:399‐408. doi:10.2147/jpr.S55598 26203274 PMC4508090

[41] Yoo H , Cho Y , Cho S . Does past/current pain change pain experience? Comparing self‐reports and pupillary responses. Front Psychol. 2023;14:1094903. doi:10.3389/fpsyg.2023.1094903 36874838 PMC9982106

[42] Mills SE , Nicolson KP , Smith BH . Chronic pain: a review of its epidemiology and associated factors in population‐based studies. Br J Anaesth. 2019;123(2):e273‐e283. doi:10.1016/j.bja.2019.03.023 31079836 PMC6676152

[43] Erlenwein J , Przemeck M , Degenhart A , et al. The influence of chronic pain on postoperative pain and function after hip surgery: a prospective observational cohort study. J Pain. 2016;17(2):236‐247. doi:10.1016/j.jpain.2015.10.013 26548971

[44] Fetz K , Lefering R , Kaske S . Pre‐trauma pain is the strongest predictor of persistent enhanced pain patterns after severe trauma: results of a single‐centre retrospective study. Medicina. 2023;59(7):1327. doi:10.3390/medicina59071327 37512138 PMC10383629

[45] Koechlin H , Coakley R , Schechter N , Werner C , Kossowsky J . The role of emotion regulation in chronic pain: a systematic literature review. J Psychosom Res. 2018;107:38‐45. doi:10.1016/j.jpsychores.2018.02.002 29502762

[46] Baliki MN , Apkarian AV . Nociception, pain, negative moods, and behavior selection. Neuron. 2015;87(3):474‐491. doi:10.1016/j.neuron.2015.06.005 26247858 PMC4529956

[47] Azizoddin DR , Schreiber K , Beck MR , et al. Chronic pain severity, impact, and opioid use among patients with cancer: an analysis of biopsychosocial factors using the CHOIR learning health care system. Cancer. 2021;127(17):3254‐3263. doi:10.1002/cncr.33645 34061975 PMC9981278

[48] Syrjala KL , Jensen MP , Mendoza ME , Yi JC , Fisher HM , Keefe FJ . Psychological and behavioral approaches to cancer pain management. J Clin Oncol. 2014;32(16):1703‐1711. doi:10.1200/JCO.2013.54.4825 24799497 PMC4031190

[49] Gorin S , Krebs P , Badr H , et al. Meta‐analysis of psychosocial interventions to reduce pain in patients with cancer. J Clin Oncol. 2012;30(5):539‐547. doi:10.1200/jco.2011.37.0437 22253460 PMC6815997

[50] Azizoddin DR , DeForge SM , Baltazar A , et al. Development and pre‐pilot testing of STAMP + CBT: an mHealth app combining pain cognitive behavioral therapy and opioid support for patients with advanced cancer and pain. Support Care Cancer. 2024;32(123):123. doi:10.1007/s00520-024-08307-7 38252172 PMC11088794

[51] Desiree R , Azizoddin RA , Kessler D , et al. Leveraging mobile health technology and research methodology to optimize patient education and self‐management support for advanced cancer pain. Support Care Cancer. 2021;29(10):5741‐5751. doi:10.1007/s00520-021-06146-4 33738594 PMC8410657

[52] Darnall BD , Ziadni MS , Krishnamurthy P , et al. “My surgical success”: effect of a digital behavioral pain medicine intervention on time to opioid cessation after breast cancer surgery‐a pilot randomized controlled clinical trial. Pain Med. 2019;20(11):2228‐2237. doi:10.1093/pm/pnz094 31087093 PMC6830264

[53] Funk‐Lawler R , Mundey KR . Understanding distress among patients with cancer receiving specialized, supportive care services. Am J Hosp Palliat Med. 2020;37(10):830‐836. doi:10.1177/1049909120905780 32066250

[54] Azizoddin DR , Allsop M , Farah S , et al. Oncology distress screening within predominately Black veterans: outcomes on supportive care utilization, hospitalizations, and mortality. Cancer Med. 2023;12(7):8629‐8638. doi:10.1002/cam4.5560 36573460 PMC10134375

[55] Porter LS , Steel JL , Fairclough DL , et al. Caregiver‐guided pain coping skills training for patients with advanced cancer: results from a randomized clinical trial. Palliat Med. 2021;35(5):952‐961. doi:10.1177/02692163211004216 33775175 PMC8265951

[56] Davis SN , Bergeron S , Sadikaj G , Corsini‐Munt S , Steben M . Partner behavioral responses to pain mediate the relationship between partner pain cognitions and pain outcomes in women with provoked vestibulodynia. J Pain. 2015;16(6):549‐557. doi:10.1016/j.jpain.2015.03.002 25827063

[57] Schmidt BL , Hamamoto DT , Simone DA , Wilcox GL . Mechanism of cancer pain. Mol Interv. 2010;10(3):164‐178. doi:10.1124/mi.10.3.7 20539035 PMC2895277

[58] Zuo L , Prather ER , Stetskiv M , et al. Inflammaging and oxidative stress in human diseases: from molecular mechanisms to novel treatments. Int J Mol Sci. 2019;20(18):4472. doi:10.3390/ijms20184472 31510091 PMC6769561

[59] Salvemini D , Little JW , Doyle T , Neumann WL . Roles of reactive oxygen and nitrogen species in pain. Free Radic Biol Med. 2011;51(5):951‐966. doi:10.1016/j.freeradbiomed.2011.01.026 21277369 PMC3134634

[60] Falk S , Bannister K , Dickenson AH . Cancer pain physiology. Br J Pain. 2014;8(4):154‐162. doi:10.1177/2049463714545136 26516549 PMC4616725

[61] Clark AK , Malcangio M . Microglial signalling mechanisms: cathepsin S and fractalkine. Exp Neurol. 2012;234(2):283‐292. doi:10.1016/j.expneurol.2011.09.012 21946268

[62] Kawasaki Y , Zhang L , Cheng J‐K , Ji R‐R . Cytokine mechanisms of central sensitization: distinct and overlapping role of interleukin‐1β, interleukin‐6, and tumor necrosis factor‐α in regulating synaptic and neuronal activity in the superficial spinal cord. J Neurosci. 2008;28(20):5189‐5194. doi:10.1523/jneurosci.3338-07.2008 18480275 PMC2408767

[63] Denk F , McMahon SB , Tracey I . Pain vulnerability: a neurobiological perspective. Nat Neurosci. 2014;17(2):192‐200. doi:10.1038/nn.3628 24473267

[64] Loprinzi CL , Lacchetti C , Bleeker J , et al. Prevention and management of chemotherapy‐induced peripheral neuropathy in survivors of adult cancers: ASCO guideline update. J Clin Oncol. 2020;38(28):3325‐3348. doi:10.1200/jco.20.01399 32663120

[65] Dorsey SG , Kleckner IR , Barton D , et al. The National Cancer Institute clinical trials planning meeting for prevention and treatment of chemotherapy‐induced peripheral neuropathy. JNCI. 2019;111(6):531‐537. doi:10.1093/jnci/djz011 30715378 PMC7962883

[66] Gewandter JS , Dworkin RH , Finnerup NB , Mohile NA . Painful chemotherapy‐induced peripheral neuropathy: lack of treatment efficacy or the wrong clinical trial methodology? Pain. 2017;158(1):30‐33. doi:10.1097/j.pain.0000000000000653 27564867 PMC6784316

[67] Colvin LA . Chemotherapy‐induced peripheral neuropathy: where are we now? Pain. 2019;160(Suppl 1):S1‐S10. doi:10.1097/j.pain.0000000000001540 31008843 PMC6499732

[68] Smith EM , Pang H , Cirrincione C , et al. Effect of duloxetine on pain, function, and quality of life among patients with chemotherapy‐induced painful peripheral neuropathy: a randomized clinical trial. JAMA. 2013;309(13):1359‐1367. doi:10.1001/jama.2013.2813 23549581 PMC3912515

[69] Loprinzi CL , Lacchetti C , Bleeker J , et al. Prevention and management of chemotherapy‐induced peripheral neuropathy in survivors of adult cancers: ASCO Guideline Update. J Clin Oncol. 2020;38(28):3325‐3348. doi:10.1200/jco.20.01399 32663120

[70] Zinboonyahgoon N , Patton ME , Chen Y‐YK , Edwards RR , Schreiber KL . Persistent post‐mastectomy pain: the impact of regional anesthesia among patients with high vs low baseline catastrophizing. Pain Med. 2021;22(8):1767‐1775. doi:10.1093/pm/pnab039 33560352 PMC8502459

[71] Echeverria‐Villalobos M , Stoicea N , Todeschini AB , et al. Enhanced recovery after surgery (ERAS): a perspective review of postoperative pain management under ERAS pathways and its role on opioid crisis in the United States. Clin J Pain. 2020;36(3):219‐226. doi:10.1097/ajp.0000000000000792 31868759

[72] Tian Y , Li Q , Pan Y . Prospective study of the effect of ERAS on postoperative recovery and complications in patients with gastric cancer. Cancer Biol Med. 2022;19(8):1274‐1281. doi:10.20892/j.issn.2095-3941.2021.0108 PMC9425188 34259423

[73] Bi Y , Ye Y , Zhu Y , Ma J , Zhang X , Liu B . The effect of ketamine on acute and chronic wound pain in patients undergoing breast surgery: a meta‐analysis and systematic review. Pain Pract. 2021;21(3):316‐332. doi:10.1111/papr.12961 33150677

[74] Mayer DK , Travers D , Wyss A , Leak A , Waller A . Why do patients with cancer visit emergency departments? Results of a 2008 population study in North Carolina. J Clin Oncol. 2011;29(19):2683‐2688. doi:10.1200/jco.2010.34.2816 21606431 PMC3139372

[75] Azizoddin DR , Wilson JM , Flowers KM , et al. Daily pain and opioid administration in hospitalized patients with cancer: the importance of psychological factors, recent surgery, and current opioid use. Pain. 2023;164(8):1820‐1827. doi:10.1097/j.pain.0000000000002880 36893325 PMC10363176

[76] Poulin PA , Romanow HC , Rahbari N , et al. The relationship between mindfulness, pain intensity, pain catastrophizing, depression, and quality of life among cancer survivors living with chronic neuropathic pain. Support Care Cancer. 2016;24(10):4167‐4175. doi:10.1007/s00520-016-3243-x 27193116

[77] Lahousse A , Roose E , Leysen L , et al. Lifestyle and pain following cancer: state‐of‐the‐art and future directions. J Clin Med. 2021;11(1):195. doi:10.3390/jcm11010195 35011937 PMC8745758

[78] Gatchel RJ , Neblett R , Kishino N , Ray CT . Fear‐avoidance beliefs and chronic pain. J Orthop Sports Phys Ther. 2016;46(2):38‐43. doi:10.2519/jospt.2016.0601 26828236

[79] Darnall BD , Roy A , Chen AL , et al. Comparison of a single‐session pain management skills intervention with a single‐session health education intervention and 8 sessions of cognitive behavioral therapy in adults with chronic low back pain: a randomized clinical trial. JAMA Netw Open. 2021;4(8):e2113401. doi:10.1001/jamanetworkopen.2021.13401 34398206 PMC8369357

[80] Hernandez Silva E , Lawler S , Langbecker D . The effectiveness of mHealth for self‐management in improving pain, psychological distress, fatigue, and sleep in cancer survivors: a systematic review. J Cancer Surviv. 2019;13(1):97‐107. doi:10.1007/s11764-018-0730-8 30635865

[81] Swartz MC , Lewis ZH , Deer RR , et al. Feasibility and acceptability of an active video game–based physical activity support group (Pink Warrior) for survivors of breast cancer: randomized controlled pilot trial. JMIR Cancer. 2022;8(3):e36889. doi:10.2196/36889 35994321 PMC9446134

[82] Roeckel L‐A , Le Coz G‐M , Gavériaux‐Ruff C , Simonin F . Opioid‐induced hyperalgesia: cellular and molecular mechanisms. Neuroscience. 2016;338:160‐182. doi:10.1016/j.neuroscience.2016.06.029 27346146

[83] Eastman P , Le BH , Grant I , Berry S . Is opioid‐induced hyperalgesia a genuine issue for palliative care patients and clinicians? J Clin Oncol. 2014;32(31_suppl):197. doi:10.1200/jco.2014.32.31_suppl.197

[84] Lee M , Silverman SM , Hansen H , Patel VB , Manchikanti L . A comprehensive review of opioid‐induced hyperalgesia. Pain Physician. 2011;14(2):145‐161. 21412369

[85] Kenter EGH , Zylicz Z . Differentiating neuropathic pain, opioid‐induced hyperalgesia and opioid tolerance; considerations following a remarkable case. Adv Palliat Med. 2010;9(3):93‐98.

[86] Bulls HW , Hamm M , Wasilewski J , Olejniczak D , Bell SG , Liebschutz JM . “To prescribe or not to prescribe, that is the question”: perspectives on opioid prescribing for chronic, cancer‐related pain from clinicians who treat pain in survivorship. Cancer. 2024;130(17):3034‐3042. doi:10.1002/cncr.35299 38567685

[87] Jones KF , Fu MR , Wood ML , et al. “It is so easy for them to dismiss”: a phenomenological study of cancer survivors with chronic cancer‐related pain. J Palliat Med. 2023;26(8):1090‐1099. doi:10.1089/jpm.2022.0538 36944115 PMC10440651

[88] O’Regan A , Fish LJ , Makarushka C , et al. Managing chronic pain in cancer survivorship: communication challenges and opportunities as described by cancer survivors. Am J Hosp Palliat Med. 2024;41(1):78‐86. doi:10.1177/10499091231164634 36927121

[89] DeForge S , Smith K , Anderson K‐A , et al. Pain coping, multidisciplinary care, and mHealth: patients’ views on managing advanced cancer pain. Psychooncology. 2024;33(2):e6308. doi:10.1002/pon.6308 38366975 PMC11071444

[90] Bulls HW , Hamm M , Wasilko R , et al. Manifestations of opioid stigma in patients with advanced cancer: perspectives from patients and their support providers. JCO Oncol Pract. 2022;18(10):e1594‐e1602. doi:10.1200/OP.22.00251 35878073 PMC9835931

[91] Bulls HW , Hamm M , Wasilko R , et al. “I refused to get addicted to opioids”: exploring attitudes about opioid use disorder in patients with advanced cancer pain and their support people. J Pain. 2023;24(6):1030‐1038. doi:10.1016/j.jpain.2023.01.015 36709854 PMC11225606

[92] Azizoddin DR , Knoerl R , Adam R , et al. Cancer pain self‐management in the context of a national opioid epidemic: experiences of patients with advanced cancer using opioids. Cancer. 2021;127(17):3239‐3245. doi:10.1002/cncr.33532 33905550 PMC8355015

[93] Azizoddin DR , Soens MA , Beck MR , Flowers KM , Edwards RR , Schreiber KL . Perioperative sleep disturbance following mastectomy: a longitudinal investigation of the relationship to pain, opioid use, treatment, and psychosocial symptoms. Clin J Pain. 2023;39(2):76‐84. doi:10.1200/jco.2020.39.28_suppl.192 36650603 PMC9968504

[94] Finan PH , Goodin BR , Smith MT . The association of sleep and pain: an update and a path forward. J Pain. 2013;14(12):1539‐1552. doi:10.1016/j.jpain.2013.08.007 24290442 PMC4046588

[95] Nijs J , Lahousse A , Kapreli E , et al. Nociplastic pain criteria or recognition of central sensitization? Pain phenotyping in the past, present and future. J Clin Med. 2021;10(15):3203. doi:10.3390/jcm10153203 34361986 PMC8347369

[96] Irwin MR , Olmstead R , Carroll JE . Sleep disturbance, sleep duration, and inflammation: a systematic review and meta‐analysis of cohort studies and experimental sleep deprivation. Biol Psychiatry. 2016;80(1):40‐52. doi:10.1016/j.biopsych.2015.05.014 26140821 PMC4666828

[97] American Cancer Society . Cancer Treatment & Survivorship Facts & Figures 2022‐2024. 2022.

[98] Glare PA , Davies PS , Finlay E , et al. Pain in cancer survivors. J Clin Oncol. 2014;32(16):1739‐1747. doi:10.1200/jco.2013.52.4629 24799477 PMC4031191

[99] Moscato S , Cortelli P , Chiari L . Physiological responses to pain in cancer patients: a systematic review. Comput Methods Progr Biomed. 2022;217:106682. doi:10.1016/j.cmpb.2022.106682 35172252

[100] Zhao J , Donovan HS , Sereika S , Campbell G . Dynamic associations between daily pain and mood during chemotherapy for gynecologic cancers. Pain Manag Nurs. 2024;25(1):56‐61. doi:10.1016/j.pmn.2023.07.002 37563052

[101] Wright JT , Herzberg MC . Science for the next century: deep phenotyping. J Dent Res. 2021;100(8):785‐789. doi:10.1177/00220345211001850 33749358 PMC8258723

[102] Robinson PN . Deep phenotyping for precision medicine. Hum Mutat. 2012;33(5):777‐780. doi:10.1002/humu.22080 22504886

[103] Edwards RR , Dworkin RH , Turk DC , et al. Patient phenotyping in clinical trials of chronic pain treatments: IMMPACT recommendations. Pain Reports. 2021;6(1):e896. doi:10.1097/pr9.0000000000000896 PMC5965275 27152687

[104] Edwards RR , Schreiber KL , Dworkin RH , et al. Optimizing and accelerating the development of precision pain treatments for chronic pain: IMMPACT review and recommendations. J Pain. 2023;24(2):204‐225. doi:10.1016/j.jpain.2022.08.010 36198371 PMC10868532

[105] Quirk DA , Johnson ME , Anderson DE , et al. Biomechanical phenotyping of chronic low back pain: protocol for BACPAC. Pain Med. 2023;24(Suppl 1):S48‐s60. doi:10.1093/pm/pnac163 36315101 PMC10403313

[106] Griffioen MA , Glutting J , O'Toole RV , et al. Transition from acute to chronic pain in lower extremity fracture patients: a pain phenotyping protocol. Nurs Res. 2020;69(2):149‐156. doi:10.1097/nnr.0000000000000407 31977841 PMC7254979

[107] Starkweather A , Ward K , Eze B , Gavin A , Renn CL , Dorsey SG . Neurophysiological and transcriptomic predictors of chronic low back pain: study protocol for a longitudinal inception cohort study. Res Nurs Health. 2022;45(1):11‐22. doi:10.1002/nur.22200 34866207 PMC8792278

